# A review on arsenic contamination in drinking water: sources, health impacts, and remediation approaches

**DOI:** 10.1039/d4ra08867k

**Published:** 2025-01-27

**Authors:** Bashdar Abuzed Sadee, Salih M. S. Zebari, Yaseen Galali, Mahmood Fadhil Saleem

**Affiliations:** a Department of Food Technology, College of Agriculture Engineering Sciences, Salahaddin University-Erbil Erbil Kurdistan Region Iraq bashdar.sadee@su.edu.krd; b Department of Animal Resource, College of Agriculture Engineering Sciences, Salahaddin University-Erbil Erbil Kurdistan Region Iraq; c Department of Nutrition and Dietetics, Cihan University-Erbil Erbil Iraq

## Abstract

Arsenic (As) contamination in groundwater has become a global concern, and it poses a serious threat to the health of millions of people. Groundwater with high As concentrations has been reported worldwide. It is widely recognized that the toxicity of As largely depends on its chemical forms, making As speciation a critical issue. Numerous studies on As speciation have been conducted, extending beyond the general knowledge on As to the toxicity and health issues caused by exposure to various As species in water. This article reviews various As species, their sources and health effects, and treatment methods for the removal of As from contaminated water. Additionally, various established and emerging technologies for the removal of As contaminants from the environment, including adsorption (using rocks, soils, minerals, industrial by-products, biosorbents, biochars, and microalgal and fungal biomass), ion exchange, phytoremediation, chemical precipitation, electrocoagulation, and membrane technologies, are discussed. Treating As-contaminated drinking water is considered the most effective approach to minimize the associated health risks. Finally, the advantages and disadvantages of various remediation and removal methods are outlined, along with their key advantages. Among these techniques, the simplicity, low cost, and ease of operation make adsorption techniques desirable, particularly with the use of novel functional materials like graphite oxides, metal–organic frameworks, carbon nanotubes, and other emerging functional materials, which are promising future alternatives for As removal.

## Abstract 2

Effective and ecofriendly remediation techniques are important for the removal of arsenic species from drinking water.

## Graphical abstract

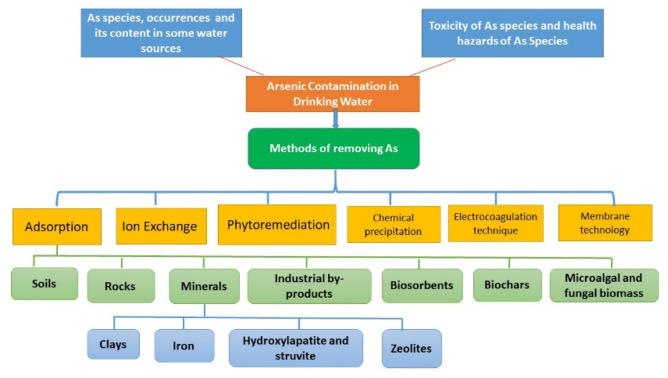

## 1 Introduction

The presence of heavy metals beyond a certain level in drinking water, and their absorption and accumulation in edible and non-edible fractions of plants, can lead to health issues in both animals and humans.^1^ Therefore, measuring the quantity of certain metallic elements is vital, as the consumption of a high quantity of these elements is destructive.^2^ The pollution caused by As, which is known to be a human carcinogen, affects hundreds of millions of people worldwide. Inorganic As (iAs) is a major contributor to the development of cancers in the skin, lungs, bladder, liver, prostate, and kidneys in humans.^3^ The water and soil of many parts of the world, especially South and Southeast Asian countries, suffer from contamination by As, affecting 100 million people worldwide, with as many as 57 million in Bangladesh alone. It is a major global concern because of the adverse impacts of As on plants, marine animals and humans.^4–8^ As finds extensive use in various sectors, including metallurgy, electronics, agriculture, and the manufacturing of chemical weapons, livestock, pesticides, fertilizers, and pharmaceutical chemicals.^5,9^ Interactions between rocks and water are the fundamental factors responsible for the liberation of As and the reduction of groundwater quality in aquifer systems.^9^

Because of its high toxicity and carcinogenicity, As is one of the major causes of environmental pollution. Vegetables can be polluted by the uptake of As from different environmental sources such as irrigation water and deposited dusts.^10^ Millions of people around the world are threatened by the exposure to As. There are many sources, such as drinking water and food products of animal and plant origin, which lead to exposure of human beings to As.^11,12^

The availability, solubility, and toxicity of different As forms depend on factors such as pH, ionic conditions, phosphorus, and other elemental contents in the environment. Additionally, differences in uptake rates affect the level of cellular exposure to As. Most of the As released into the environment is inorganic and tends to accumulate by binding to organic soil matter.^13^ Elemental speciation is a well-recognized discipline within analytical chemistry. As is a widespread element in the environment, introduced through both natural processes and human activities.^5^ Seafood and seaweed are the main dietary sources of total As for human beings, predominantly in the form of organic As (oAs) species. Nevertheless, there are exclusion where high quantities of iAs have been detected, such as in the edible seaweed Hijiki (*Hizikia fusiformis*), freshwater fish, and blue mussels.^14^ Under aerobic conditions, arsenate (As^V^) is the predominant form, while under anaerobic conditions, arsenite (As^III^) is more common. The higher concentration of As^III^ in paddy fields due to waterlogging and the presence of As in rice, which is a potential As^III^ accumulator plant, raise significant concerns.^13^

The environmental As contamination is mainly caused by human activities, which poses a serious threat to millions of people. These individuals face life-threatening complications from consuming water contaminated with As or food grown in As-tainted soils or irrigated with As-laden water. Researchers and authorities have recognized As contamination as a critical issue from the twentieth to the twenty-first century.^15,16^

This paper aims to review and provide updates on health consequences of various As species and the latest technological advancements in arsenic removal methods, exploring the potential of these innovations to address the issue of As contamination in groundwater.

## 2 As occurrences

Human exposure to high levels of As is frequently connected to drinking water. The chemical form and degree of methylation of As have substantial impacts on its toxicity, bioaccumulation, and mobility.^17,18^ It is more typical for As in groundwater to have a geogenic origin globally than an anthropogenic one. Strongly decreasing aquifers that are typically formed from alluvium and inland or closed basins in dry or semi-arid regions are common places to find high geogenic levels of As in groundwater. Slow groundwater flow and the presence of geologically young sediments are characteristics of both habitats.^19^ As concentrations in groundwater are also influenced by hydrogeochemical factors such pH, dissolved organic carbon, and competing anions. Additionally, geothermal regions and places with historical mining activity, where sulfur dioxide minerals are typically oxidized and have As-rich groundwaters.^20^

Groundwater's As contents and the specific As species exist depending on different factors including As sources, redox conditions, groundwater flushing, the bioavailability of organic matter, and the partition of clay and peat layers.^20–24^ There is evidence that long-term droughts increase the concentration of As in drinking water, despite the fact that this toxin's primary source is geology. Additional human activities that can lead to the contamination of aquifers and surface water include mining As.^25^ These activities increase the content of As in the environment, and it is thought that the source may be parent rocks and nearby mountains. As mobility linked to anthropogenic activities may also occur in soils and groundwater, especially under anaerobic conditions.^26^ The solubility of As and other minerals is enhanced by oxidation-reduction reactions, which leads to an increase in their mobility in the environment *via* the water system. Several factors including aquifer characteristics, grain size, organic content, oxidation–reduction processes, adsorption–desorption, precipitation–dissolution, and biological activity influence this mobility.^27^

## 3 As species

**Fig. 1:**
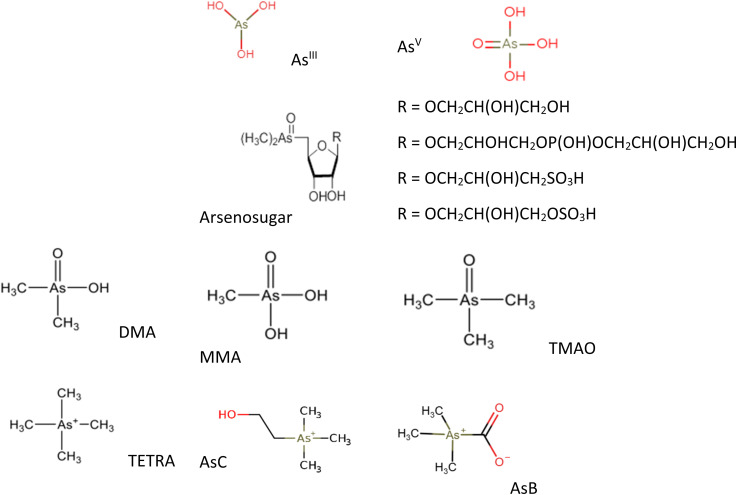
Chemical structure of the most common As species.

There are numerous oAs and iAs species with different toxicity characteristics. In the natural environment, As is mostly found in the oxidation states of −3, +1, +3, and +5. It is rarely found in the elemental (neutral) state.^5,28^ As primarily exists in the form of the inorganic species As^III^ and As^V^. In nature, As typically appears in combination with sulfur, oxygen, and iron.^27^ In general, elevated concentrations of As in groundwater have been observed under reducing conditions. In oxic environments such as surface water, As mainly exists as As^V^ in the form of oxyanions H_2_AsO_4_^−^ (pH 3–6) and HAsO_4_^2−^ (pH 8–10). Under anoxic conditions, like those found in floodplain groundwater, As^III^ species primarily exist as neutral molecular forms (H_3_AsO_3_) at pH levels ≤ 9.2.^29,30^ As a result, the negatively charged As^V^ is more likely to be adsorbed onto sediments, while the adsorption of As^III^ occurs more slowly due to its neutral charge.^31^ In addition to As^III^ and As^V^, there are other methylated derivatives of As compounds of environmental significance, such as arsenobetaine (AsB) and arsenocholine (AsC) and arsenosugars (As-sugars). Methylated As compounds are present in marine ecosystems as a result of the enzymatic methylation of iAs, leading to compounds with 1–4 methyl groups.^32,33^ In marine organisms, iAs has the potential to undergo bioconversion into methylated species such as MMA or AsB.^34^ AsC acts as a metabolic precursor for AsB in aquatic animals. AsB is formed when labeled AsC is incorporated, along with smaller amounts of iAs, monomethylarsonic acid (MMA), and/or dimethylarsinic acid (DMA).^35^ It is thought that the breakdown of arsenosugars results in the production of non-toxic AsC, which is predominantly found in aquatic animals.^36^Fig. 1 provides the examples of some common As species.

## 4 Toxicity of As species and health hazards

A major issue is the global presence of As in its natural or geogenic form, which has a wide range of negative health effects on both people and wildlife. Additionally, As-contaminated water enhances the presence of iAs in the diet. As shows different levels of toxicity in mammals depending on various factors, including its form (organic or inorganic), valence state, absorption rate, elimination process, solubility, and particle size.^37^ Chronic exposure to trivalent As is considered to be of greater toxic potential than the pentavalent form. However, the claimed higher toxicity of trivalent As remains a subject of debate, as organic arsenicals are generally considered less toxic than inorganic forms. It is worth mentioning that methylated organic arsenicals such as MMA and DMA could be potentially less toxic than iAs.^5,6^ In mammals, iAs undergoes metabolism into methylated metabolites. Initially, this methylation was thought to be a detoxification mechanism, but the discovery of more toxic methylated trivalent metabolites in human urine proved that this process is opposed. The trivalent methylated metabolites of iAs, monomethylarsonous acid (MMA^III^) and dimethylarsinous acid (DMA^III^), have been demonstrated to be more acutely toxic than their precursor compounds.^38,39^

Different iAs species including As^III^ and As^V^ are recognized as carcinogens.^40,41^ Conversely, oAs species such as MMA and DMA are deemed less toxic than iAs but are still classified as cancer-inducing agents. In contrast, AsC and AsB are identified as non-toxic As species.^42^ iAs is dangerous and offers no beneficial metabolic role; it can lead to skin diseases, circulatory and neurological disorders, and even cancer.^43^ Additionally, As-contaminated water contributes to the presence of iAs in food, making dietary consumption a major exposure pathway. Prolonged exposure to water with high levels of iAs (>100 μg L^−1^) is linked to the development of non-melanoma skin cancer, as well as lung and bladder cancers.^44^

Exposure to As can pose harmful effects on both humans and other organisms. In the case of acute As toxicity, symptoms such as nausea, vomiting, and severe diarrhea may occur.^45,46^ Inorganic As poisoning has been associated with a wide range of health issues including various cancers (bladder, lung and kidney), respiratory and immune system disorders, endocrine disruption, reproductive health issues, neurological conditions, liver disease, gastrointestinal disturbances, genotoxic effects, arsenicosis, and skin infections and cancer.^47,48^ Furthermore, chronic toxicity is linked to more serious health outcomes, disease, diabetes, digestive disturbances, high blood pressure, cardiovascular problems and gangrene.^45,46^ The trivalent oxidation state of As is linked to increased potency as a cytotoxin and clastogen, potentially triggering harmful biological pathways that contribute to gastrointestinal disorders, cancers,^49–51^ and genotoxic, mutagenic, and carcinogenic impacts.^52^

**Fig. 2:**
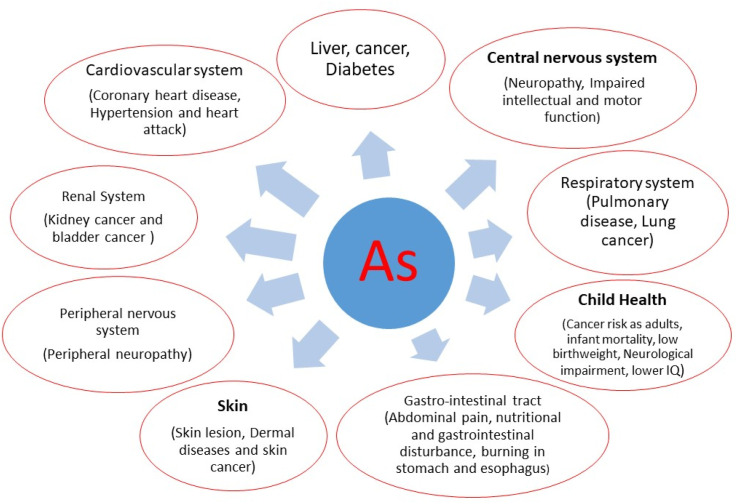
Some widespread diseases in humans caused by exposure to As 4. As in drinking water.

The severity of As poisoning largely depends on factors such as the amount of exposure, the nutritional status of individuals, the duration of exposure, and immune response. Furthermore, long-term exposure to As is particularly linked to skin conditions such as arsenicosis, which is a global health concern, not limited to any specific country.^53^ As is genotoxic because it hinders the repair of damaged DNA, making it a carcinogen.^54^ Epigenetic changes are among the most researched mechanisms of As poisoning. Experimental studies have also shown that As can cause epigenomic alterations even in healthy individuals.^55^ Scientific research, supported by empirical evidence, indicates that As adversely affects neurodevelopment and causes birth defects, even at low levels of exposure during early life.^44^ As exposure during pregnancy has been linked to alterations in gene expression pathways associated with diabetes, increasing the likelihood of developing the disease in adulthood.^56^ More details of the consequences of As exposure are presented in Fig. 2.

The maximum amount of As that food should contain has been recommended by numerous international organizations. This is because As's substantial enrichment and biotransformation have a detrimental effect on human health. Since it is poisonous to humans, it can negatively impact individuals of any age or health condition. iAs is the class of As that has the greatest potential for toxicity. As contamination of groundwater has been reported in numerous countries across the world, including the United States, China, Taiwan, Mexico, Mongolia, Myanmar, Argentina, India, Chile, Cambodia, Nepal, the Philippines, Vietnam, Afghanistan, Indonesia, and Pakistan (Table 1). However, the situation in Bangladesh is particularly severe. It is estimated that between 35 and 77 million people in Bangladesh are chronically exposed to high levels of As through their drinking water, with the levels significantly exceeding the WHO guideline of 10 μg L^−1^. A national survey in Bangladesh revealed that As concentrations above 50 μg L^−1^, as well as those between 10 and 50 μg L^−1^, contribute to over 24 000 adult deaths each year.^53^ As primarily exists in water as As^III^ and As^V^.^70^ The level of As contamination in groundwater varies by geographic region, and it has been proven that there is a link between As concentrations and human activities.^60^ The concentrations of As in unpolluted fresh and sea water are <1 to 10 μg L^−1^ and from 1 to 3 μg L^−1^, respectively.^71^

**Table 1:** As concentration in water in some countries around the world

As concentration (μg L^−1^)	As source	Sampling location	Reference
0.19–7.8	Groundwater (drinking water)	Sulaimani and Erbil-Iraq	57
1.06	Groundwater (drinking water)	Akre, Duhok-Iraq	58
0.58	Groundwater (drinking water)	Dokan, Sulaimani-Iraq	59
141	Tube wells	Murshidabad district-West Bengal, India	60
260–730	Groundwater	Nadia district-West Bengal, India	61
15–1300	Groundwater	Kandal, Cambodia	62
1.25–5114	Shallow groundwater	Southern Thailand	63
<1.0–850	Groundwater	South Vietnam	64
1.3	Drinking water	Central China	65
0.5–278	Shallow groundwater	Michigan, USA	66
<0.5–10	Groundwater	Baseline, UK	67
<1.0–80	Groundwater	Southwest, England	68
48 810	Groundwater	Chapai-Nawabganj, Bangladesh	69

One of the earliest studies on the As content of tube well water in Bangladesh revealed the presence of As in the water samples. The investigation tested water from 3490 tube wells, finding that 28.1% contained As levels exceeding 50 μg L^−1^ and 21.9% had concentrations between 10 and 50 μg L^−1^. Subsequently, a comprehensive nationwide As survey conducted by the Bangladesh Geological Survey (BGS) and the Department of Public Health Engineering (DPHE) reported that 27% of shallow tube wells had As concentrations above the national drinking water standard of Bangladesh (50 μg L^−1^).^69^ A geological survey conducted by the United States estimated that the average As concentration in groundwater is around 11 μg L^−1^ or lower. However, groundwater aquifers in the western United States were found to have significantly higher As concentrations.^72^ A study has been conducted to estimate the As concentration in ground water in Kurdistan region, Iraq, which found that the concentration of As ranged from 0.19 to 7.8 μg L^−1.^^57^ Meanwhile, in Akre-Duhok and Dokan-Sulaimani, groundwater which is used as drinking water was found to have As concentrations of 1.06 (ref. 59) and 0.58 μg L^−1^,^58^ respectively.

## 5 Methods for removing As

In groundwater, As primarily exists as As^III^ and As^V^ in varying proportions. Removing As^III^ is more complicated than As^V^, so a pretreatment step, specifically oxidizing As^III^ to As^V^, is necessary to enhance the effectiveness of most As removal methods. This is especially crucial for anaerobic groundwater, where As^III^ is the dominant form of As.^73^ In recent years, extensive research has been conducted on As removal techniques to enhance the removal of As from the environment. The different technologies available for the removal of As include adsorption, ion exchange, precipitation, phytoremediation, coagulation–flocculation, and membrane technologies. The studies on the approaches and up-to-date modifications that have been performed to address As contamination in water are presented in Table 2. In experiments on As removal, various methods are employed, each with its own advantages and disadvantages. The effectiveness of these methods is detailed in Table 2.

**Table 2:** Overview of the methods used for As removal from water

Method	Types	As removal	Advantages	Disadvantages	Reference
Adsorption	Rocks, soils, minerals, industrial by-products, biosorbents, biochars and microalgal and fungal biomass	95%	Safe operation, easy handling, flexibility, cost-effective, sludge-free, and high removal efficiency	Sorbents require replacement once the adsorption bed becomes saturated and exhausted, eventually losing its separation capacity. They lack self-monitoring capabilities and have a low specific surface area when metal oxides are used. Additionally, they are only suitable for wastewater with low arsenic concentrations	74–78
Ion exchange	Natural polymeric materials or synthetic organic substances	97.9% (pH: 3.5–7)	Complete removal and recovery of metal substances, with minimal production of toxic sludge	Requires regular regeneration to maintain full removal efficiency; expensive; each exchanger is specific to a particular As species; has an unfavorable selectivity order; the resin is more reactive to natural anions; lowering the pH, which may lead to potential corrosion problems	74 and 79–81
Phytoremediation	Innovative approaches and plants (phytobial, phytoextraction, phytostabilization, phytofiltration, and phytovolatilization, nanophytoremediation)	99.9%	High quality, efficiency and effective for aquatic system; environmentally friendly and economically valuable; preventing the spread of contaminants in land restoration	The most cost-effective treatment methods, widely accepted socially across the globe; a time-consuming process; climate and tropical zones impact many hyperaccumulating plants; microbes generate additional toxic substances; lacks widespread applications; hazardous pollutants interfere with the plants' metabolic processes, hindering their growth and development	82–86
			Nanophytoremediation enhances the efficiency of phytoremediation, supports *in situ* remediation, boosts the degradation of pollutants into less toxic forms, and is cost-effective		
Chemical precipitation	Reagents such as Fe salts, sulfides, mg, and Ca salts	95%	Straightforward and efficient; targets specific components for removal	Consistently forms silt; associated with high processing costs	87–90
Electrocoagulation technique	FeCl_2_; FeSO_4_; Al_2_(SO_4_)_3_	99.9%	A new and promising approach for As removal in drinking water; efficient, cost-effective, easy to maintain, and operates with locally available materials	Ineffective for extracting As^III^; generates contained sludge with high energy consumption; highly influenced by the form and dose of coagulants, solution pH, and the presence of other competing anions	91–94
Membrane technology	Microfiltration (MF), nanofiltration (NF), ultrafiltration (UF), and reverse osmosis (RO)	96%	Excellent efficiency, low energy consumption, and superior filtration performance; applicable for various separation methods	High costs and significant water rejection	95–97

### 5.1 Adsorption

Adsorption is both efficient and cost-effective compared to the other As removal methods. It is an appropriate technology for As treatment in developing countries with unreliable electricity and a shortage of skilled personnel. Adsorbents with over 95% efficiency have been reported for removing As^III^ and As^V^. Unlike other methods, adsorption generally does not require the addition of chemicals.^98^ The effectiveness of this process primarily relies on van der Waals forces and electrostatic attraction between the adsorbed molecules.^99^ It is important to note that the efficiency of this method is influenced by factors such as exposure time, pH levels, the presence of other chemical species, adsorbent dosage, initial As concentration, and temperature.

#### 5.1.1 Rocks

Soils and volcanic rocks, due to their abundance and local availability, can serve as cost-effective adsorbents for As.^100^ Among the most common volcanic rocks are pumice and scoria, typically found in regions with young volcanic fields. However, both pumice and scoria have shown relatively low efficiency in removing oxyanions such as As^III^ and As^V^ from water. Pristine scoria achieved a removal efficiency of just 14% for As^III^ at a pH of 5.0,^101^ while raw pumice was able to remove less than 20% of As^V^ from an acidic solution with a pH of 3.^102^ Rocks from the Soyatal formation in Mexico were utilized for treating As-contaminated water. In laboratory tests, this clay-rich limestone demonstrated superior As remediation compared to rocks from the El Abra/Tamaulipas and Las Espinas formations. The calcareous shale of the Soyatal formation, which contains As-adsorbing minerals such as illite and kaolinite, could serve as an effective low-cost remediation option. Additionally, rocks from the Zimapan region of Mexico successfully removed As (at 0.6 mg L^−1^) from water.^103^

#### 5.1.2 Soils

Different types of soils were applied for As removal from water. Termite mound which is mainly composed of silicon (Si), iron (Fe), aluminum (Al), and titanium oxide (TiO_2_) was used to remove As from water with removal efficiency of 13.5 mg g^−1^ for As^V^ at a pH of 7.0. Termite mounds showed the high As^V^ adsorption capacity at 13.5 mg g^−1^. This is likely because As has a strong affinity for iron and Al_2_O_3_.^104^ Laterite soil, Sewage irrigated soil and natural red earth were also administrated to remove As from water with efficiencies of 1.38, 0.37 and 0.02 mg g^−1^ for As^III^ at pH 5.7,^105^ 7.5 (ref. 106) and 5.5,^107^ respectively. Laterite soil and natural red earth were able only to remove 0.04 and 0.013 mg per g As^V^, respectively. The As removal ability of laterite soil is linked to its Fe and Al content. In general, raw laterite samples with higher levels of Fe and Al showed greater As adsorption capacities. The removal of As^III^ from aqueous solutions by red soil (laterite) involves both direct adsorption of As^III^ and the oxidation of As^III^ to As ^V^ prior to adsorption. Previous studies have indicated that around 20–25% of As^III^ can be oxidized to As^V^ during the adsorption process by laterite or red soil.^105^

#### 5.1.3 Minerals

##### 5.1.3.1 Clays

Clay minerals primarily consist of hydrous Al silicates, along with small amounts of Fe, magnesium (Mg), and other cations. Clays are widely abundant in both aquatic and terrestrial environments. Their large surface areas make them capable of adsorbing various metal species.^108^ Clays containing oxides and hydroxides, act as potential adsorbents for As removal.^109^ Clay materials have also been demonstrated as sorbents to remove As^III^ and As^V^ anions from contaminated water, though their effectiveness is highly dependent on pH levels. As^V^ adsorption on clay minerals is highest at low pH and generally decreases as the pH rises above 5, while As^III^ adsorption follows a parabolic pattern, reaching its peak around pH 8.5.^110^ Geological materials such as gibbsite, goethite, hematite, Fe-coated zeolites, laterite, limestone, and oxisols, and both montmorillonite and bentonite clays have been utilized to remove As from synthetic and real environmental waters.^111,112^

Natural or modified clays including metakaoline, clinoptilolite and synthetic zeolite, bentonite, natural clay, smectite, Fuller's earth and montmorillonite had capacity to adsorb As^V^ from different media at different pH values as follows: 10–22.5,^113^ 1.48,^114^ 0.25–0.75,^115^ 0.16,^116^ 91.42 (ref. 117) and 7.22–15.15 (ref. 118 and 119) mg g^−1^, respectively. Meanwhile, bentonite, Fuller's earth and montmorillonite showed the capacity to remove As^III^ as follows: 0.82–7.3,^114,120^ 50.08 (ref. 117) and 11.36–16.58 (ref. 119) mg g^−1^, respectively.

##### 5.1.3.2 Iron

Iron oxy-hydroxides found in sediments led to As immobilization through sorption and co-precipitation.^121^ Iron exists as Fe(ii) and Fe(iii) in various mineral forms, including oxides, hydroxides, and oxy-hydroxides. Most Fe oxide minerals, such as goethite (α-FeOOH), hematite (α-Fe_2_O_3_), and magnetite (Fe_3_O_4_), are thermodynamically stable in natural systems, while others, such as ferrihydrite (Fe_5_HO_8_·4H_2_O) and maghemite (γ-Fe_2_O_3_), are considered intermediate forms.^122^ The removal of As using sediments rich in Fe minerals was studied.^123^ Due to the abundance of Fe oxide minerals in nature, they are considered a low-cost alternative for household water treatment. Previous research has shown that As^III^ and As^V^ can be effectively adsorbed onto amorphous Fe oxide.^124^ Additionally, abundant clay minerals like illite and kaolinite, as well as Fe-rich laterite and sediments, were evaluated as effective adsorbents for As remediation in northwest Argentina.^125^ The use of Fe oxides for As removal from water is a well-established method. However, separating As-loaded fine particles from the treated water poses a challenge. Beyond oxides, certain iron carbonate minerals such as siderite (FeCO_3_) have also been used to treat As-contaminated water.^126^

##### 5.1.3.3 Hydroxylapatite and struvite

Hydroxylapatite (Ca_5_(PO_4_)_3_OH) is a common mineral formed in wastewater systems and it effectively adsorbs As under near-neutral to acidic conditions. Furthermore, struvite (MgNH_4_PO_4_·6H_2_O) is a mineral found naturally in geochemical and biological environments, and frequently precipitates during wastewater treatment processes.^127,128^ A study evaluated the potential for As adsorption onto struvite and hydroxylapatite at pH levels between 8 and 11, using solutions containing 2.7–0.125 mM phosphate and 0.05 mM of either As^III^ or As^V^. Over a period of 7 days, As^III^ removal was minimal, while As^V^ removal improved with increasing pH. The highest removal efficiency, reaching 74%, was observed in pH 11 solutions containing struvite. This finding highlights that struvite is particularly effective for treating As^V^-contaminated water under alkaline conditions, unlike most traditional adsorbents that are effective only under acidic or neutral conditions.^128^

##### 5.1.3.4 Zeolites

Zeolite minerals primarily consist of aluminosilicates with a three-dimensional framework of AlO_4_ and SiO_4_ tetrahedra. These are interconnected by sharing oxygen atoms, creating a network of cages and channels. These cavities hold mobile water molecules and exchangeable cations, such as alkali or alkaline earth metals, which contribute to the mineral's ability to perform ion exchange and adsorption processes.^129^ Lanthanum-loaded zeolite has a higher capacity for As^V^ adsorption compared to activated alumina and activated carbon; however, it is more expensive.^130^ Modifying zeolite with MnO_2_ significantly enhances its amphoteric properties, reducing the equilibrium pH when the initial solution pH is alkaline. This MnO_2_ treatment improves the zeolite's As^V^ removal efficiency across a wide pH range (4.0–9.0),^131^ making it practical for real-world applications by eliminating the need for acid/base adjustments. Additionally, zeolites have been modified with cationic surfactants such as hexadecyltrimethylammonium (HDTMA) and ethylhexadecyldimethylammonium (EHDDMA).^132^ The As adsorption behaviors of these zeolites are summarized in Table 3.

**Table 3:** As adsorption efficiency of natural/modified clays

Zeolites	Condition	Capacity			Reference
		As^III^	As^V^	Total As	
Natural zeolites	200 μg L^−1^ of water treated with HCl	—	—	75%	133
Chabazite-phillipsite, clinoptilolite, and volcanic glass	Deionized water was used to prepare the solution spiked with 100 μg L^−1^	—	—	40–78%	134
Clinoptilolite	Zeolites were washed with 2 M HCl and the pH was adjusted at 5, water solution contained 500 μg per L As	98%	98%	—	135
Cancrinite	Loading cancrinite alumina to water at pH range of 4.9–7.0	—	—	34.5 mg g^−1^	136
Natural zeolite	Aqueous solution contained 5 mM As, zeolite loaded with lanthanum at pH range 2–8	—	95%	—	130
Zeolite P	Exchange of zeolite sodium with cerium(iii) at pH 3–10		23.42 mg g^−1^		137
Raw zeolite	Iron-coated zeolite at a pH range of 3.0–10.0		0.68 mg g^−1^		138
Zeolite	Coating zeolite with magnetic nanoparticle (γ-Fe_2_O_3_) at a pH value of 2.5		44 mg g^−1^		139
Clinoptilolite-Ca	Modification of clinoptilolite-Ca zeolite with MnO_2_		2.5 μg g^−1^		131
Zeolite	Groundwater spiked with 2000 μg L^−1^, magnetic nanoscale Fe–Mn binary oxides loaded zeolite at pH 7		99%		140
Clinoptilolite	Zeolite (clinoptilolite) supported mono- (Fe or Al) and bi-metallic (Fesingle bondAl) oxides at pH 5		3.86 mg g^−1^		141

#### 5.1.4 Industrial by-products

Industrial wastes such as sludge, ash, and red mud have been applied for the treatment of As-contaminated water. Table 4 presents the adsorption capacities of various industrial wastes. While some of these wastes were only effective in removing As^V^ from water, other materials such as acid mine drainage sludge and iron-rich sludge showed greater effectiveness in removing As^III^ compared to As^V^.

**Table 4:** Adsorption capabilities of several industrial waste materials for As

Adsorbents	Condition	Capacity (mg g^−1^)		Reference
		As^III^	As^V^	
Acid mine drainage sludge (AMDS)	The maximum removal efficiencies of As on AMDS under a pH of 7.0	58.5	19.7	142
Aluminum-based adsorbent (ABA) and coal mine drainage sludge coated polyurethane (CMDS-PU)	The adsorbents were efficient at pH range (3–10)		10–31	143
Magnetic bio-sludge (MS) containing activated sludge and magnetite (Fe_3_O_4_) nanoparticles	MS featured a macroporous structure with a surface area of 78 m^2^ g^−1^ and a pore volume of 0.53 cm^3^ g^−1^, pH 2.6 at 25 °C		18.5	144
Goethite and calcite	The adsorption of As^V^ is highest under acidic pH conditions, while As^III^ achieves maximum adsorption at neutral to slightly basic pH levels	66.9	21.5	145
Fe-based backwashing sludge (FBBS)	At pH 7 to pH 10, the removal of As ^V^ was enhanced with an increase in ion strength (0.01–1 M NaNO_3_)	59.7	43.32	146
Red mud-modified biochar produced from rice straw	pH of 2 for As ^V^ and pH of 10 for As^III^	0.52	5.923	147
Biochar prepared from pinewood and natural hematite	γ-Fe_2_O_3_ particles on the carbon surface served as sorption sites, pH of 7		0.429	148
Agrowaste derived biochars	Impregnate ZnO on biochar derived from agricultural residual biomass, pH of 6.00–6.50		25.9	149
Waste rocks	Particle size of 45–75 μm, mole ratio of 1.6 for OH^−^ to modification salts, aging time of 72 h, liquid/solid ratio of 63, 25 °C. pH of 7		5.99	150

**Fig. 3:**
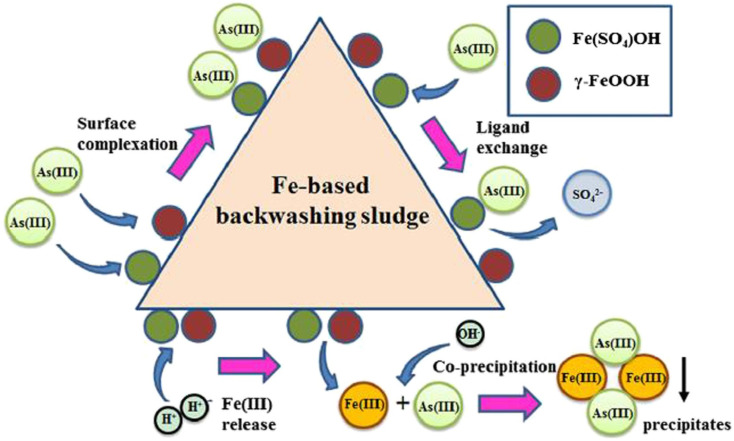
Suggested mechanisms for removing As^III^ using FBBS as an adsorbent, with permission from Wu *et al.* 2013.^151^

Sludge from certain industrial processes, such as Fe-based backwashing sludge (FBBS), generated during Fe(ii) removal processes, is effective for As treatment. FBBS has rough surfaces and a high Brunauer–Emmett–Teller (BET) surface area of 148.41 m^2^ g^−1^. It primarily consists of sulfate-interlayered Fe hydroxide [Fe(SO_4_)OH], ferric oxyhydroxide (γ-FeOOH), quartz (SiO_2_), and calcium carbonate (CaCO_3_). The removal mechanism for As^V^ mainly involves the formation of inner-sphere complexes, while As^III^ adsorption probably occurs through ligand exchange, coprecipitation with Fe(iii), and adhesion to surface hydroxyl groups, as illustrated in Fig. 3.^146,151^

#### 5.1.5 Biosorbents

**Fig. 4:**
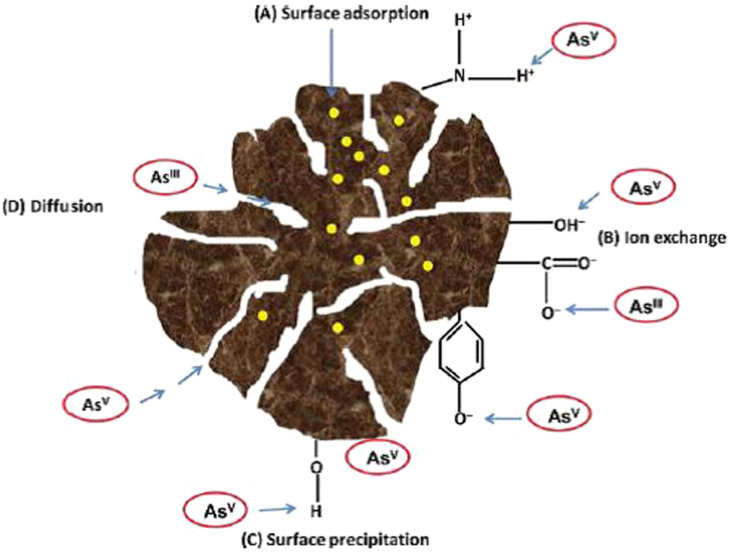
Illustration of the mechanism of arsenic sorption on the surface of a biosorbent.^152^

Biosorption can also remove As^III^ and As^V^ from water. This process involves the use of non-living biomass to bind and eliminate As *via* physicochemical reactions. Biosorbents typically contain functional groups such as hydroxyl (–OH), carboxyl (–COOH), phenolic, amino (–NH_2_), sulfhydryl (–SH), alcoholic, and ester groups. These groups are highly effective at removing As from water *via* mechanisms such as sorption, complexation, ion exchange, diffusion, or co-precipitation, as illustrated in Fig. 4. Biosorption is regarded as an environmentally friendly alternative to traditional methods such as ion exchange, precipitation, membrane filtration, reverse osmosis, and electrodialysis.^152^

Cellulose modified with copper (Cell-N–Cu) proved to be effective for removing As^V^ from water.^153^ Similarly, cellulose beads loaded with iron oxyhydroxide (BCF) were capable of removing both As^III^ and As^V^ from aqueous solutions, with relatively high adsorption capacities of 99.6 mg g^−1^ and 33.2 mg g^−1^, respectively, at a pH of 7.0.^154^ Alginate is a polysaccharide typically extracted from brown algae and certain bacteria, including *Azotobacter vinelandii* and various *Pseudomonas* species. Hydrous iron oxide-impregnated alginate beads (HIO-alginate beads),^155^ calcium alginate/activated carbon composite beads,^156^ acid mine drainage sludge (AMDS) treated alginate beads, goethite alginate beads, and pure alginate beads^157^ are significant adsorbents for treating As-contaminated water. Treated alginate beads demonstrated high adsorption capacities for both As^III^ (18.25 mg g^−1^) and As^V^ (21.79 mg g^−1^), which is attributed to the significant presence of amorphous ferric hydroxide. Additionally, Mn in acid mine drainage sludge can oxidize As^III^ to As^V^ on the treated alginate bead surfaces, aiding in the removal of As^III.^^157^ However, immobilizing hydrous iron oxide-impregnated alginate beads onto alginate beads reduces the adsorption capacity of hydrous iron oxide. The As adsorption capacities of hydrous iron oxide-alginate beads (47.8 mg g^−1^ for As^III^ and 55.1 mg g^−1^ for As^V^) were lower than that of unimpregnated hydrous iron oxides, which had capacities of 393.7 mg g^−1^ for As^III^ and 200.4 mg g^−1^ for As^V^.^155^

Chitosan, an alkaline-deacetylated derivative of chitin, is widely used due to its high hydrophilicity, abundance of hydroxyl and amino groups that serve as active adsorption sites, non-toxicity, natural abundance, biocompatibility, and biodegradability. In its natural form, chitosan is soft, prone to agglomeration, and tends to form gels, making its specific binding sites less accessible for sorption.^158^ However, there are some limitations to using chitosan as a metal recovery agent, such as its low acid stability and poor mechanical properties. To address this, chitosan has been modified in various ways to enhance its physical and chemical properties, improving its As removal efficiency. For example, chitosan has been processed into beads^159^ immobilized in sodium silicate,^160^ complexed with transition metal ions such as copper (Cu(ii)), Fe(iii), lanthanium (La(iii)), molybdenum (Mo(vi)), and zirconium (Zr(iv)), coated on ceramic alumina,^158^ immobilized on pumice,^102^ and impregnated with molybdate.^161^ Chitosan has attracted growing attention as a renewable polymeric material for the treatment of water and wastewater contaminated with metals^162^ especially As.^163,164^ A summary of different chitosan adsorbents is presented in Table 5.

**Table 5:** Different bio-adsorbents for As removal

Bio-adsorbents	pH	Isotherm model fit	Capacity (mg g^−1^)		Reference
			As^III^	As^V^	
Chitosan gel beads modified with molybdate	2–3	Langmuir	70	230	161
Coating natural biopolymer, chitosan, on ceramic alumina, using a dip-coating process	4	Langmuir, Freundlich	56.50	96.46	158
Alumina nanoparticles and immobilized them in chitosan-grafted polyacrylamide matrix	7.2	Freundlich	—	6.56	165
Chitosan zerovalent iron nanoparticles (CIN)	7	Langmuir	94	119	166
Xanthated chitosan granules (XCB)	7.5	Langmuir, Freundlich	48	36	166
Chitosan-graphene oxide-gadolinium oxide	3–7	Langmuir, Freundlich	—	252.12	167
Ultrafine nanobiosorbent of cerium modified chitosan	8	Langmuir	57.5	—	168
Iron–chitosan composites	7	Langmuir	16.15	22.47	169
α-Fe_2_O_3_ impregnated chitosan	5	Langmuir	9.36	—	170
TiO_2_-impregnated chitosan bead without exposure to UV light	6.61–7.02	Langmuir	2.2	2.05	171
TiO_2_-impregnated chitosan bead without exposure to UV light	6.61–7.02	Langmuir	6.4	4.93	171
Granular chitosan-titanium	8	Langmuir	—	165.6 a	172
Magnetic chitosan-based composite microparticles	—	—	33.68	34.61	173

a μg L^−1^.

#### 5.1.6 Biochars

Biochar is a diverse carbon material containing various surface functional groups, created through the thermal transformation of different waste materials. Typically, biochar is a carbon-rich substance produced when biomass, such as wood, leaves, manure, or municipal waste sludge, is heated in a closed system with limited or no air. Biochar derived from various waste sources is widely used for removing organic and inorganic pollutants, including heavy metals.^174^ Oxygen-rich functional groups can make biochar surfaces negatively charged, particularly in their pristine state. As a result, significant removal of anions and oxyanions occurs at low pH (below 4.0), where biochar's surface functional groups become protonated.^175^ Oxyanions such as arsenate undergo complex, pH-dependent speciation. Under alkaline conditions, the negatively charged biochar surfaces are less effective as As adsorbents.^176^ However, engineered biochars have been successfully used to remove As^III^ and As^V^ from water.^177^

Both biochar and modified biochars have proven effective for arsenic remediation.^174,178^ Several approaches have been explored to modify biochar and improve its As sorption, including biochar modified with colloidal and nano-sized oxyhydroxides,^179^ biochar impregnated with Fe,^180^ and biochar infused with Fe/Mn oxides.^181^ Biochars derived from oak bark, oak wood, pine bark, and pine wood successfully removed As.^182^ Pre-treating biomass with AlCl_3_ followed by slow pyrolysis at 600 °C for 1 hour creates a biochar/AlOOH nano-flake nanocomposite,^183^ which was highly effective for As removal, with a Langmuir adsorption capacity of around 17.41 mg g^−1^. Additionally, magnetic biochar with abundant γ-Fe_2_O_3_ particles on its surface showed an As^V^ adsorption capacity of 3.15 mg g^−1^.^184^ For instance, biochar was produced from pinewood and treated with nanoscale zerovalent iron (nZVI). The resulting nZVI-supported biochar (nZVI/BC) showed a high capacity for As^V^ removal across a wide pH range (3–8).^179^ Additionally, a magnetic biochar was synthesized by pyrolyzing a mixture of naturally occurring hematite mineral and pinewood biomass. The hematite-modified biochar demonstrated a significantly higher ability than that of unmodified biochar to remove As^V^ from water, which is attributed to γ-Fe_2_O_3_ particles on the carbon surface acting as sorption sites *via* electrostatic interactions.^148^ A nano-zero valent zinc (nZVZn), biochar (BC)/nZVZn and BC/hydroxyapatite-alginate (BC/HA-alginate) composite was developed for the removal of iAs species from water. A high percentage of As^III^ and As^V^ removal was achieved by nZVZn at pH 3.4 (96% and 94%, respectively) compared to BC/nZVZn (90% and 88%) and BC/HA-alginate (88% and 80%) at pH 7.2. The Freundlich model provided the best fit for the sorption data of As^III^ and As^V^ across all sorbents, particularly for nZVZn.^185^

#### 5.1.7 Microalgal and fungal biomass

Microalgae and fungal biomass are some of the most extensively studied biosorbents due to their potential for heavy metal removal applications. The biosorption capacity of dead green algae (*Maugeotia genuflexa*) biomass for removing As^III^ from aqueous solutions has been explored, achieving a maximum monolayer sorption capacity of 57.48 mg g^−1^ at pH 6.^186^ It has been documented that *Lessonia nigrescens* has been used as a biosorbent for As^V^ removal from aqueous solutions.^187^ The fungal biomass of *Aspergillus niger* was coated with iron oxide, which resulted in maximum removals of 95% for As^V^ and 75% for As^III^ at pH 6.^188^ Pretreating fungal (Mycan) biomass with cationic surfactants such as hexadecyltrimethylammonium bromide (HDTMA-Br) and dodecylamine (DA) also improved the biosorption efficiency. The maximum adsorption capacity was 57.85 mg g^−1^ for Mycan/HDTMA biomass and 33.31 mg g^−1^ for Mycan/DA biomass, significantly higher than the unmodified biomass's capacity for As^V^ (24.52 mg g^−1^).^189^

### 5.2 Ion exchange

The medium used for ion exchange typically consists of resins made from natural polymeric materials or synthetic organic substances containing ionic functional groups that facilitate the exchange process. Strong and weak acid resins are used to exchange cations, while strong and weak base resins are used for anions. Since As is present in water as an anion, weak base resins are employed. Groundwater is passed through a resin-packed column, following filtration to prevent suspended particulates from entering and clogging the column.^190^

The U.S. Environmental Protection Agency (EPA) has recommended specific ion exchange resins, specifically those in chloride form, for the elimination of As.^191^ Customized anion exchangers that can reduce As concentrations to below 10 μg L^−1^ are available.^192^ Synthetic ion exchange resins typically contain quaternary ammonium groups and a polystyrene cross-linked with divinylbenzene as the polymeric matrix. These resins are particularly effective for adsorbing As^V^.^193^ Competing ions can impact the efficiency of the ion exchange process, and exhausted resin is regenerated using an aqueous NaCl solution.^192^ The uptake of As^V^ is not influenced by pH or the concentration of the influent. However, for the removal of As^III^, oxidation is necessary when using ion exchange resins, as neutral H_3_AsO_3_ is present, and H_2_AsO_3_^−^ is only available at pH levels above 8.^193^ The resin surface is preloaded with chloride ions through HCl pretreatment. These chloride ions are readily exchanged with As^III^, As^V^, or other anions such as SO_4_^2−^, F^−^, and NO_3_^−^. The As level in the water, the type of ion-exchange resin used, high sulfate salt, total dissolved solids and the presence of competing ions are key factors influencing the effectiveness of As removal.^194^ Regeneration water and spent resin with a high As content require additional treatment before they can be disposed of or reused. Alternatively, the resins can be used in a disposable, non-renewable ion exchange process.

A research group successfully reduced As levels below the permissible limit of 10 μL^−1^ using a Hybrid Ion Exchange/Electrodialysis (IXED) method.^195^ Similarly, a study conducted the removal of As^V^ ions from water using a laboratory-scale IXED system. They found that the reduction in As^V^ concentration in the solution passing through the ion exchange bed closely matched the test data. However, the results from the resin-free compartments for As^V^ showed variations of 6% and 16% towards the end of the study. These discrepancies were attributed to assumptions in the model, particularly the influence of unaccounted As^V^ ion species.^196^ A study evaluated the performance of two full-scale ion exchange (IX) systems, one point-of-entry (POE) reverse osmosis (RO) system, and nine point-of-use (POU) RO units for the simultaneous removal of As and several co-occurring contaminants from drinking water. Conducted as part of the U.S. Environmental Protection Agency's As Treatment Demonstration Program, the IX systems, equipped with strong base anionic (SBA) resins, effectively reduced As to levels below their respective maximum contaminant limits of 10 μg per L As.^79^

### 5.3 Phytoremediation

**Fig. 5:**
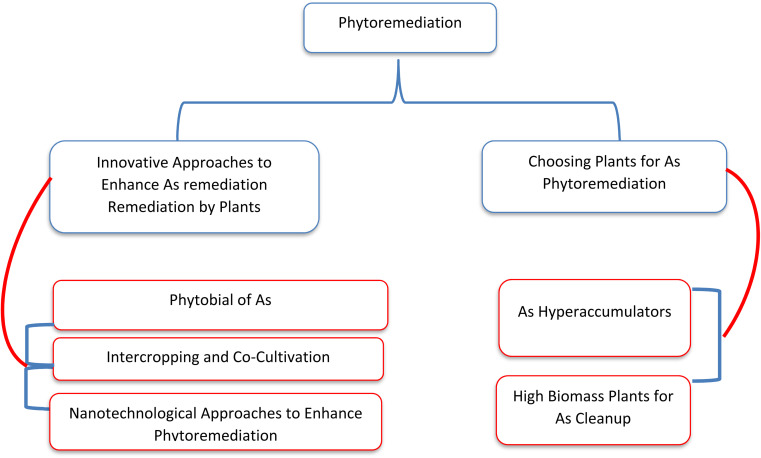
Different approaches for As phytoremediation.

Phytoremediation (phyto–plant, remediation–clean) involves the use of green plants to remove pollutants from contaminated environments. This method offers several advantages, primarily being an autotrophic system with large biomass that requires minimal nutrient input. It is easy to manage and widely accepted due to its aesthetic appeal and environmental sustainability.^197^ A summary of phytoremediation of As using different plants and approaches is presented in Fig. 5. Some plant species use transporter proteins to move metals, while others absorb them directly from the aquatic environment through water uptake, which leads to adsorption and accumulation of metals in the plant's aerial parts. Phytoremediation is categorized into different methods—phytoextraction, phytostabilization, phytofiltration, and phytovolatilization—based on the pathways for metal uptake and transport within the plants.^198^ Hyperaccumulating plants possess a high remediation potential due to their exceptional ability to tolerate and manage heavy metals in their tissues. The efficiency of phytoremediation can be improved through assisted or induced methods, such as the use of chelators or the inoculation of microbes, to enhance metal removal.^199^ Hyperaccumulator plants are capable of accumulating metals in their shoots above a specific threshold, which is 1000 mg kg^−1^ for As.^200^ Additionally, the bioaccumulation factor, which indicates the transfer of metal from soil to plant, and the translocation factor, which reflects the transfer of metal from the root to the shoot, are used to classify a plant as a hyperaccumulator.^201^ For a plant to be considered an As hyperaccumulator, both the bioaccumulation factor and the translocation factor values must exceed one. Various approaches of phytoremediation for As are presented in Fig. 5.

As hyperaccumulation has predominantly been observed in fern species belonging to the *Pteris* genus, including *Pteris vittata*,^201^*P. longifolia*,^202^*P. quadriaurita*, *P. cretica*, *P. ryiunkensis*,^203^ and *Pityrogramma calomelanos*.^204^ Additionally, a plant from the Brassicaceae family, Isatis cappadocica, has also demonstrated As hyperaccumulation capabilities. The fern species Pteris vittata (Brake fern) has shown significant success in As remediation. This fast-growing, easily propagated, and perennial fern can accumulate up to 22 grams of As per kilogram of its biomass, which is 200 times more than any other known species. It also accumulates As very rapidly, capable of removing As from concentrated solutions (500 μg L^−1^) within just two days. Additionally, the fern can be reused repeatedly for As removal.^205^*Pteris vittata* was employed in a hydroponic system without mechanical aeration. The approach was simple, with the plants' rhizomes positioned above the water surface and nutrients provided in minimal amounts to promote root growth (achieving root lengths of 500 mm in four months). Starting with varying initial water As concentrations of 50 μg L^−1^, 500 μg L^−1^, and 1000 μg L^−1^, the *Pteris* plants were able to reduce As levels to 10 to 0.1 μg L^−1^ within 1–5 days, 4–6 days, and 8–10 days, respectively.^206^

High biomass aquatic plants such as *Ceratophyllum demersum*,^207^*Hydrilla verticillata*,^208^*Lemna gibba*,^209^*Lemna minor*,^210^*Azolla caroliniana*,^211^*Pistia stratiotes*,^212^*Salvinia natans*,^213^ and *Eichhornia crassipes*^214^ can subject in the remediation of contaminated water bodies. *Lemna gibba* has been shown to acquire As from contaminated surface water containing 41.37–47 g L^−1^ As for up to 1022 mg per kg dry biomass in 21 days. The biomass accumulation and As removal capacity of *Lemna gibba* have been reported to be as high as 73.6 t per ha per year and 752 kg As per ha per year, respectively. In another study, *Eichhornia crassipes* accumulated around 498 mg As per kg dry weight from a solution containing 0.5 mg L^−1^ of arsenic over 10 days, reducing the initial arsenic concentration by 83%. Hydrilla verticillata removed up to 72% of arsenic from an 8 L solution (1500 μg per L As) within 45 days, with a maximum As concentration of 388 μg per g dry weight. These fast-growing plants with high biomass accumulation are easy to harvest, can reestablish themselves, and require minimal input for growth. They also show high tolerance to wastewater. The water fern *Micranthemum umbrosum* has been studied for As and cadmium remediation, while emergent aquatic plants such as *Cyperus vaginatus* and *Vetiveria zizanioides* have also shown effectiveness in phytoremediation studies. With a high-biomass moderate As accumulator, As removal per year can be greater than that achieved by a low-biomass hyperaccumulator.

Phytobial remediation is an innovative approach that combines the use of plants and microbes to address As contamination in the environment. Recently, plant growth-promoting bacteria (PGPB) have attracted considerable attention due to their role in enhancing phytoremediation. These bacteria not only improve the plant's tolerance to metals but also support plant growth, aiding in the large-scale removal of As.^215^ Microbes, particularly those from the rhizosphere, have been shown to help in phytoremediation, leading to growing interest in rhizoremediation as a method for reclaiming As-contaminated environments. Researchers have identified several As-resistant microorganisms that help reduce As toxicity and promote plant growth by facilitating its mobilization and accumulation in plants.^216^ Microorganisms involved in phytobial remediation employ biostimulation, bioaccumulation, and biotransformation processes to manage heavy metals. For example, *Lysinibacillus* species found in the rhizosphere of *Pteris vittata* can withstand high levels of As, tolerating up to 1136 mg L^−1^ for As^III^ and 3256 mg L^−1^ for As^V^. Furthermore, they can accumulate As, with reported capacities of 5.65 mg L^−1^ for As^III^ and 23.43 mg L^−1^ for As^V^, demonstrating their potential in reducing As toxicity in contaminated environments. Thus, plant-microbe interactions represent an eco-friendly and effective approach that accelerates the process of phytoremediation by enhancing the efficiency of contaminant removal through the synergistic effects of both plants and microbes.^82^

Intercropping is a common agricultural technique where two different crops are grown together to enhance soil conditions, improve nutrient availability, and boost soil enzyme activity.^217^ In studies, intercropping As hyperaccumulator *Pteris vittata* with As-sensitive, non-accumulator plants has shown promise in reducing arsenic contamination and mitigating As stress on companion crops. For instance, intercropping *P. vittata* with *Panax notoginseng*,^218^*Morus alba*,^219^ and maize (*Zea mays*)^220^ has been investigated for improving overall plant health and reducing field As levels. The combined or sequential use of aquatic plants has been shown to improve As removal efficiency compared to using a single plant. A study tested the successive application of three aquatic plants (*Lemna*, *Hydrilla*, and *Ceratophyllum*) in a medium containing 2500 μg L^−1^ of As over 21 days, with each plant used for 7 days. The results indicated that the highest arsenic removal (27% in 21 days) was achieved with the succession of *Hydrilla*–*Ceratophyllum*–*Lemna*.^221^ A combination tool of *Ceratophyllum demersum* and *Lemna* minor was obtained, which removed 4365 μg in 30 days from an As-supplemented medium (2500 μg L^−1^).^222^

Nanophytoremediation (NP) is an environmentally friendly technology that merges phytoremediation and nanotechnology to remediate polluted environments.^223^ This approach uses innovative nanomaterials with unique properties to enhance the removal of toxic substances from contaminated soils and water, particularly those polluted with heavy metals. By reducing the need for extensive treatment and minimizing cleanup time, NP offers a more efficient solution for addressing environmental contamination.^224^ Although research on As removal through nanophytoremediation is limited, existing studies indicate that integrating nanomaterials with plants and microbes is still in the exploratory phase. This approach shows promise in significantly improving traditional bioremediation methods.^225^ Integrating nanotechnology with phytoremediation offers numerous benefits, including enhanced decontamination efficiency for soils and water polluted with heavy metals. This combination leverages the unique properties of nanomaterials to improve the effectiveness of plant-based remediation strategies.^223^ Research indicated that salicylic acid-based NPs enhance As remediation in *Isatis cappadocica*,^226^ while the application of nano-zinc (Zn) improves As stabilization in *Helianthus annuus*.^227^ The use of NPs enhances As phytoremediation while decreasing As bioaccumulation in crops. Recent studies have indicated that applying 1000 mg L^−1^ of nano-TiO_2_ reduced As accumulation in rice by 40–90%,^228^ and at 4000 mg L^−1^,^229^ it lowered As phytotoxicity in *Vigna radiata*. Additionally, ZnO amendment promoted rice seedling growth, decreased As accumulation in roots and shoots, and increased phytochelatin levels.^230^

### 5.4 Chemical precipitation

Chemical precipitation is a method that uses reagents such as Fe salts, sulfides, Mg, and Ca salts to remove heavy metals, including As, from wastewater. These reagents convert dissolved As into low-solubility compounds, often stabilizing solid waste as well. Common techniques include forming calcium arsenate and ferric arsenate. However, calcium arsenate can be unstable, forming calcium carbonate and As acid in the presence of water and CO_2_, while ferric arsenate's stability varies between amorphous and crystalline forms depending on environmental conditions.^87^

The precipitation of Fe(iii) was used to enhance the removal of As^V^ from alkaline leaching solutions. This method improved the overall removal efficiency by optimizing the Fe-to-As ratio, which led to better precipitation of As^V^.^89^ Magnetite nanoparticles made from Fe(ii)/nitrate solutions are used to remove As from contaminated water.^231^ In As removal using lead oxide in an aqueous chloride solution, precipitates form, including lead hydroxide, nitrate, and oxide, which play a role in the arsenic removal process. The highest concentration of As^V^ in the As–Pb precipitates was 0.2 mg L^−1^ over a wide pH range (1.9–12.3) and various leaching times (1–48 hours). Washing the precipitates with dilute HNO_3_ improves their stability. Lead oxide proves to be an effective reagent for As removal in such solutions. In a study on As^V^ removal, the efficiency of different lead-based reagents was compared. Lead powder removed 26.5% of As^V^, while lead oxide, lead hydroxide, and lead nitrate achieved removal efficiencies of >99.9%, 98.5%, and 96.3%, respectively, in a Pb/As molar ratio of 2.12.^87^

### 5.5 Electrocoagulation technique

It has also been discovered that coagulation is a useful approach for removing As from groundwater and soil.^232^ Electrocoagulation (EC) is a multifaceted process that relies heavily on the chemistry of the aqueous medium. It utilizes electrochemical methods to generate coagulants *in situ*, based on the demand. Through the application of electrical energy, metals such as Al and Fe dissolve, destabilizing colloidal particles. This leads to the flocculation and flotation of contaminants, allowing for their efficient removal. The process is highly dependent on the interplay between the solution's properties and the electrochemical reactions.^92^ For such processes, only pre-oxidation and pH adjustment are required, simplifying the treatment. If the water's characteristics are suitable, the extraction method can bypass the sedimentation step, further streamlining As removal without the need for complex pre-treatment procedures.^233^ EC is an effective method for eliminating As^V^ and As^III^ from water, achieving removal efficiencies of 93% to 99.9%.^234^Table 6 presents the data on various coagulants used for As removal, along with their respective percentages.

**Table 6:** As removal from water of different origins *via* electrocoagulation

No	Sample	Coagulation agent	Conditions	Removal efficiency%		Reference
				As^III^	As^V^	
1	Water	Fe	Electrocoagulation: pH = 6–8	99	99	235
2	Water	Fe	Chemical coagulation; pH = 6 for As^III^; pH = 6–7 for As^V^	98	99	235
3	Water	Fe^3+^ + Al^3+^	Electrocoagulation; pH = 4–10, As removal = 1–1000 μg mL^−1^	—	—	236
4	Natural ground water	Cu–Cu	Electrocoagulation: pH = 6–7	99.89	—	237
5	Natural ground water	Zn–Zn	Electrocoagulation; pH = 6–7	99.56	—	237
6	Wastewater	Stainless steel	Electrocoagulation; As^III^ is oxidized to As^V^; pH = 5.2		86–99.6	91
7	Groundwater	Al and Fe scrap anodes	Electrocoagulation; As^III^ is oxidized to As^V^; pH = 5–8	—	93.5	238
8	Drinking water	Graphene oxide-manganese ferrite (GMF)	Adsorptive mixed matrix membrane; pH = 4	—	102^a^	239
9	Drinking water	adsorptive mixed matrix membrane	MMMs incorporated with different loading of hydrophilic GMF nanomaterial (0.5–2.0 wt%)	—	75.5 a	239

a mg g^−1^

### 5.6 Membrane technology

Membranes possess unique surface characteristics such as pore size, permeability, hydrophobicity, roughness, and dimensions, as well as separation capabilities (permeation and selectivity) due to their structure and composition. They should demonstrate resistance to chemical and mechanical stress, high permeability and selectivity, durability, and affordability. Additionally, all membrane-based processes produce a concentrated stream in which ions from the feed are collected.^240^ It has the ability to reduce As contamination in groundwater by 96%. The membrane removes As from contaminated water without retaining it and blocks microbes from passing through. The membrane is designed to allow easy disposal of contaminants and is simple to maintain, requiring minimal operational effort and no chemicals.^95^ Previous studies reported that the oxidation of As^III^ to As^V^ was undesirable due to the potential damage to the membrane.^97^ However, recent research highlights the use of various membrane types for As removal in water systems, including MF,^96^ NF,^241^ UF,^242^ and RO.^99^

A “loose” nanofiltration membrane was used to investigate the separation of As from groundwater. The membrane's molecular mass cut-off and pore size were determined through saccharide retention calculations, while its electro-kinetic surface charge was characterized using streaming potential measurements, all conducted prior to the As extraction tests.^243^ In NF and RO, the driving force is generated by applying pressure to the feed side, allowing water to pass through a hydrophilic membrane, thereby removing contaminants such as. Additionally, atmospheric pressure thermally regulated membrane processes, such as Membrane Distillation (MD), have been proven effective in treating As-contaminated water. MD is recognized for producing high-quality water with high retention rates of harmful substances, including As and other heavy metals found in groundwater. Geothermal energy, as well as geothermal water itself, can be used as a fresh water source through the MD process, making it suitable for low-cost heat sources. MD slightly reduces As elimination for both As^III^ and As^V^.^244^

A novel adsorptive membrane was developed for As removal by modifying the porous support layer of a membrane created through phase inversion. Iron oxide (Fe_3_O_4_) microspheres were embedded in the membrane's support layer using reverse filtration, followed by dopamine polymerization. These adsorptive membranes could pave the way for innovative approaches to As removal from water, while ensuring the safety of drinking water.^245,246^ The ceramic hollow fiber membrane was manufactured using low-cost kaolin, achieving a high permeate flux of 28 kg m^−2^ h for As^III^ and 25 kg m^−2^ h for As^V^ with 100% As rejection at a feed temperature of 60 °C. This met the required maximum contamination level of 10 ppb. Additionally, As concentrations up to 1000 ppm at different pH levels were tested, and no As was detected in the permeate.^247^

## 6 Conclusion

The extent of As contamination requires further investigation, as it is now known to be far more widespread than previously understood, with higher concentrations and smaller particulates found in drinking water and food samples. As affects millions of people, making it a serious global issue due to its harmful effects on plants, marine life, and humans. Due to its high toxicity and carcinogenicity, iAs is a major environmental pollutant, posing serious dangers with no beneficial metabolic function. It can cause skin diseases, circulatory and neurological disorders, and even cancer. The concentration of As in groundwater and the specific As species present depend on various factors including the sources of As, redox conditions, groundwater flow, the availability of organic matter, and the distribution of clay and peat layers. Mitigating As exposure can help reduce effects such as skin lesions and decrease the risk of cancer. However, challenges remain despite the availability of various techniques including their potential negative environmental impact. These issues must be addressed for these methods to become a more effective and influential solution for reducing health hazards.

Various methods have been reported to achieve this objective. It examined various natural and anthropogenic sources, such as adsorption (including rocks, soils, minerals, industrial by-products, biosorbents, biochars, microalgal and fungal biomass), ion exchangers, phytoremediation, chemical precipitation, electrocoagulation and membrane technologies. Treating As-contaminated water and soil is probably the most effective way to reduce health risks. Various strategies are being employed to achieve this, but many of these methods have significant drawbacks, and their by-products may contribute to secondary arsenic contamination. Therefore, to effectively address the As threat and sustainable by-products with less toxicity, new technologies, including potential hybrid solutions, are needed.

## Data Availability

The data supporting this article have been included within the article.

## Author contributions

Bashdar Abuzed Sadee: supervision, writing – original draft, conceptualization, validation, methodology. Salih M. S. Zebari: writing – review & editing, visualization, validation, Yaseen Galali: writing – review & editing, investigation, Mahmood Fadhil Saleem: writing – review & editing.

## Conflicts of interest

There is no conflict of interest to declare.
